# Bridging pro-inflammatory signals, synaptic transmission and protection in spinal explants in vitro

**DOI:** 10.1186/s13041-018-0347-x

**Published:** 2018-01-15

**Authors:** M. Medelin, V. Giacco, A. Aldinucci, G. Castronovo, E. Bonechi, A. Sibilla, M. Tanturli, M. Torcia, L. Ballerini, F. Cozzolino, C. Ballerini

**Affiliations:** 1 Department of Life Sciences, University of Trieste, 34127 Trieste, Italy; 2 International School for Advanced Studies (SISSA/ISAS), 34136 Trieste, Italy; 3 Department NEUROFARBA, University of Florence, 50139 Florence, Italy; 4 Department of DSBSC, University of Florence, 50134 Florence, Italy; 5 Department of DMSC, University of Florence, 50134 Florence, Italy

**Keywords:** Organotypic spinal slices, Network activity, Cytokines, Neuroinflammation, Neuroprotection, NGF-mimetic

## Abstract

Multiple sclerosis is characterized by tissue atrophy involving the brain and the spinal cord, where reactive inflammation contributes to the neurodegenerative processes. Recently, the presence of synapse alterations induced by the inflammatory responses was suggested by experimental and clinical observations, in experimental autoimmune encephalomyelitis mouse model and in patients, respectively. Further knowledge on the interplay between pro-inflammatory agents, neuroglia and synaptic dysfunction is crucial to the design of unconventional protective molecules. Here we report the effects, on spinal cord circuits, of a cytokine cocktail that partly mimics the signature of T lymphocytes sub population Th1. In embryonic mouse spinal organ-cultures, containing neuronal cells and neuroglia, cytokines induced inflammatory responses accompanied by a significant increase in spontaneous synaptic activity. We suggest that cytokines specifically altered signal integration in spinal networks by speeding the decay of GABA_A_ responses. This hypothesis is supported by the finding that synapse protection by a non-peptidic NGF mimetic molecule prevented both the changes in the time course of GABA events and in network activity that were left unchanged by the cytokine production from astrocytes and microglia present in the cultured tissue. In conclusion, we developed an important tool for the study of synaptic alterations induced by inflammation, that takes into account the role of neuronal and not neuronal resident cells.

**Electronic supplementary material:**

The online version of this article (10.1186/s13041-018-0347-x) contains supplementary material, which is available to authorized users.

## Introduction

Inflammatory mechanisms have been closely linked to the pathogenesis of heterogeneous diseases of the Central Nervous System (CNS), including multiple sclerosis (MS), Alzheimer’s disease (AD), amyotrophic lateral sclerosis (ALS) and Parkinson’s disease (PD) [1, 2]. In these pathologies, inflammatory cytokines (CKs) can be either delivered by activated microglia and astrocytes (CNS resident cells) or by peripheral immune cells able to infiltrate the CNS parenchyma (lymphocytes, neutrophils and mast cells). CKs release affects neurons and synapses, contributing to gray matter pathology. In experimental multiple sclerosis the harmful action of microglia on synaptic activity is mediated by tumor-necrosis factor-alfa (TNF-α) and interleukin-1beta (IL-1β), and pro-inflammatory conditions in general have been reported to tune post-synaptic NMDA and AMPA glutamate receptors, enhancing excitatory transmission and inhibiting the GABAergic one [3–5]. These observations have led to the awareness that multiple sclerosis pathophysiology, traditionally viewed as a genuine white matter autoimmune disorder with only secondary neurodegenerative components [6], involves diffuse synaptic dysfunction and loss, i.e. synaptopathy, that concurrently with demyelination contributes to grey matter atrophy. Inflammatory-dependent synaptopathy, reviewed by Mandolesi et al. (2015), has been detected in MS patients, representing a novel and promising target for future therapies [7].

Nerve growth factor (NGF), extensively studied as neuro-protector agent in neurodegenerative diseases [8], is involved in neuronal survival and reparative processes. NGF has been reported to confer CNS protection in experimental autoimmune encephalomyelitis (EAE) [9]. Recently, Xu et al. (2016) described the neuroprotective effects of a molecule (T-006) that mimic NGF activities and potentiates NGF-protection against glutamate-induced excitotoxicity [10]. In accordance to these strategies, the strongest rationale behind mesenchymal stem cell (MSC) transplantation as an effective therapeutic approach in MS, AD, PD and ALS, resides also in MSCs ability to secrete neurotrophic factors, preventing neuronal damage induced by the inflammatory insult [11]. In the development of novel therapies, the design of trophic molecules able to cross the blood brain barrier (BBB) and thus to directly target neurons, shielding them from synaptic alterations, is a timely placed issue.

Mechanistic studies of the interplay between the release of CKs, the activation of microglia, the emergence of synaptic dysfunction and the role of novel protective molecules, may require sophisticated in vitro models tested in the laboratory to investigate CNS responses at synaptic resolution.

Organotypic slice cultures developed from the embryonic mouse spinal cord represent a complex in vitro model where sensory-motor cytoarchitecture, synaptic properties and spinal cord resident cells are retained in a 3D–fashion [12, 13]. By the use of this model, we have recently shown that in the SOD1^G93A^ mouse, a genetic ALS model, spinal synapses retain greater GABA and glycine co-release than in the wild types and these changes influenced synaptic integration [14].

Here, we further exploit organotypic cultures from the embryonic mouse spinal cord to monitor the emergence of synaptopathy in pre-motor circuits following CKs transient exposure, and to test the neuroprotective efficacy of NGF-mimetic molecule MT2 [15].

We monitored synaptic activity by patch-clamp recording of visually identified ventral interneurons. Spinal cultured tissue exposed to a cocktail of pro-inflammatory CKs displayed a significant increase in spontaneous synaptic activity characterized by a speeding up of the decay phase of GABAergic inhibitory currents, that may affect temporal precision at post-synaptic site and synaptic control of network excitability. These changes were accompanied by significant production of cytokines and chemokines, astrogliosis and microglia activation. Although these inflammatory features were untouched by MT2 applications, this drug reverted all synaptic changes, suggesting the need of specific neuro-protective strategies during chronic inflammation in the CNS.

## Results

### Organotypic spinal cord cultures express TrkA and TrkB receptors

**Fig. 1:**
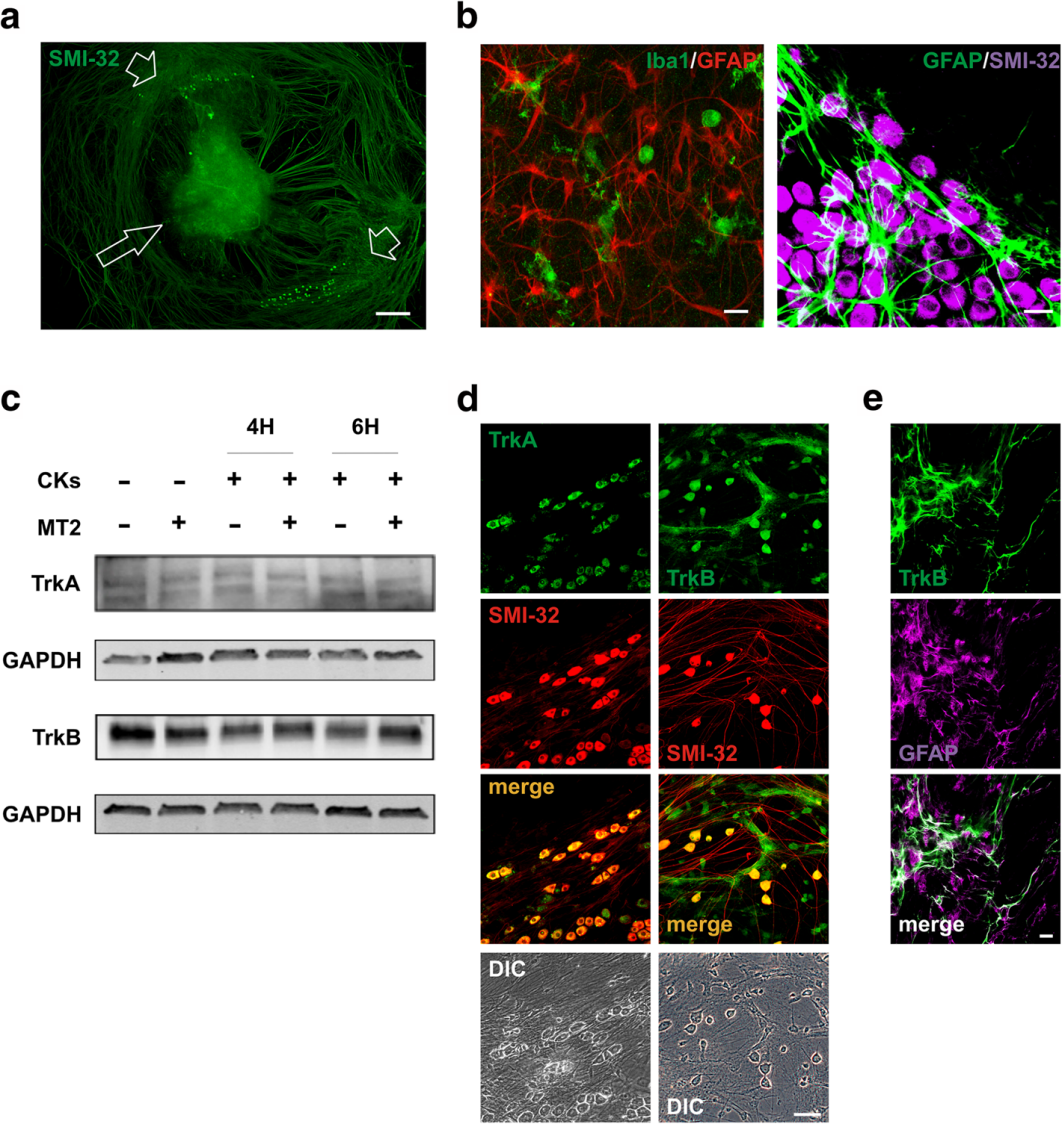
TrkA/B receptor expression in organotypic spinal cord cultures. **a** Low magnification immunofluorescence for SMI32 (green) of an organotypic spinal cord slice cultured for 2 weeks. The arrow indicates the ventral fissure, localizing the ventral horns. Note the co-cultured DRGs (arrow heads). Calibration bar 500 μm. **b** High magnification immunofluorescence labeling for microglia, astrocytes, and neurons, details of the ventral horn (left) or DRG (right) are shown. Left) green: Iba1 (microglia); red: GFAP (astrocytes). Right) green: GFAP (astrocytes); magenta: SMI-32 (neurons). Calibration bar 20 μm. **c** Western blot analysis of Tropomyosin receptor kinases A and B (TrkA and TrkB) expression in organotypic cultures stimulated with a cocktail of pro-inflammatory CKs (TNF-α, IL-1β and GM-CSF, 10 ng/ml each) for 4 or 6 h in the presence or in the absence of NGF-mimetic molecule MT2 (10 μM). GAPDH was used as house-keeping gene. Each well of the electrophoresis gel was loaded with an equal amount of proteins extracted from a pull of *n* = 5 organotypic slices. Note that TrkA and TrkB expression is not affected by treatments. This experiment is representative of 3 independent ones. **d** Immunofluorescence confocal and differential interference contrast (DIC) images for TrkA and TrkB expression in DRG neurons. Green: TrkA (left panel), TrkB (right panel). Red: SMI-32. Yellow: merge. Calibration bar 50 μm. **e** Immunofluorescence confocal images for TrkB and GFAP in the ventral horns. Green: TrkB; magenta: GFAP Calibration bar 20 μm

Organotypic spinal slices represent a biological model useful for studying the dynamics of intra-segmental processes that evidently rely on resident neuroglial cells, propriospinal neurons and circuits [12–14]. To investigate the impact of inflammation on spinal synaptic networks we used co-cultured mouse DRG and spinal cord explants after 2 weeks of in vitro growth (Fig. 1a). These cultures contain heterogeneous cell populations belonging to the neuronal and neuroglial phenotypes [12], including GFAP-positive astrocytes and Iba1-positive microglia (Fig. 1b). We exploited this model to evaluate the NGF-mimetic molecule MT2 ability to prevent synaptic modulation brought about by neuroinflammation, the latter induced by incubating (for 4 and 6 h) the tissue with pro-inflammatory CKs (TNF-α, IL-1β and GM-CSF, 10 ng/mL) [16]. Since MT2 is a ligand of TrkA and TrkB receptors, we first checked the expression of these receptors in the cultured slices. Figure 1c shows the western blot analysis obtained under basal conditions and upon CK stress in the presence or in the absence of MT2 (10 μM). We found that both receptors are expressed by organotypic cultures, and the levels of expression were comparable in all the experimental conditions. By immunofluorescence labeling (Fig. 1d) we observed that TrkA and TrkB are mainly expressed by DRG neurons and with TrkB, in part, by astrocytes (Fig. 1e).

### NGF-mimetic MT2 counteracts CKs induced increase in synaptic activity in organotypic slices

**Fig. 2:**
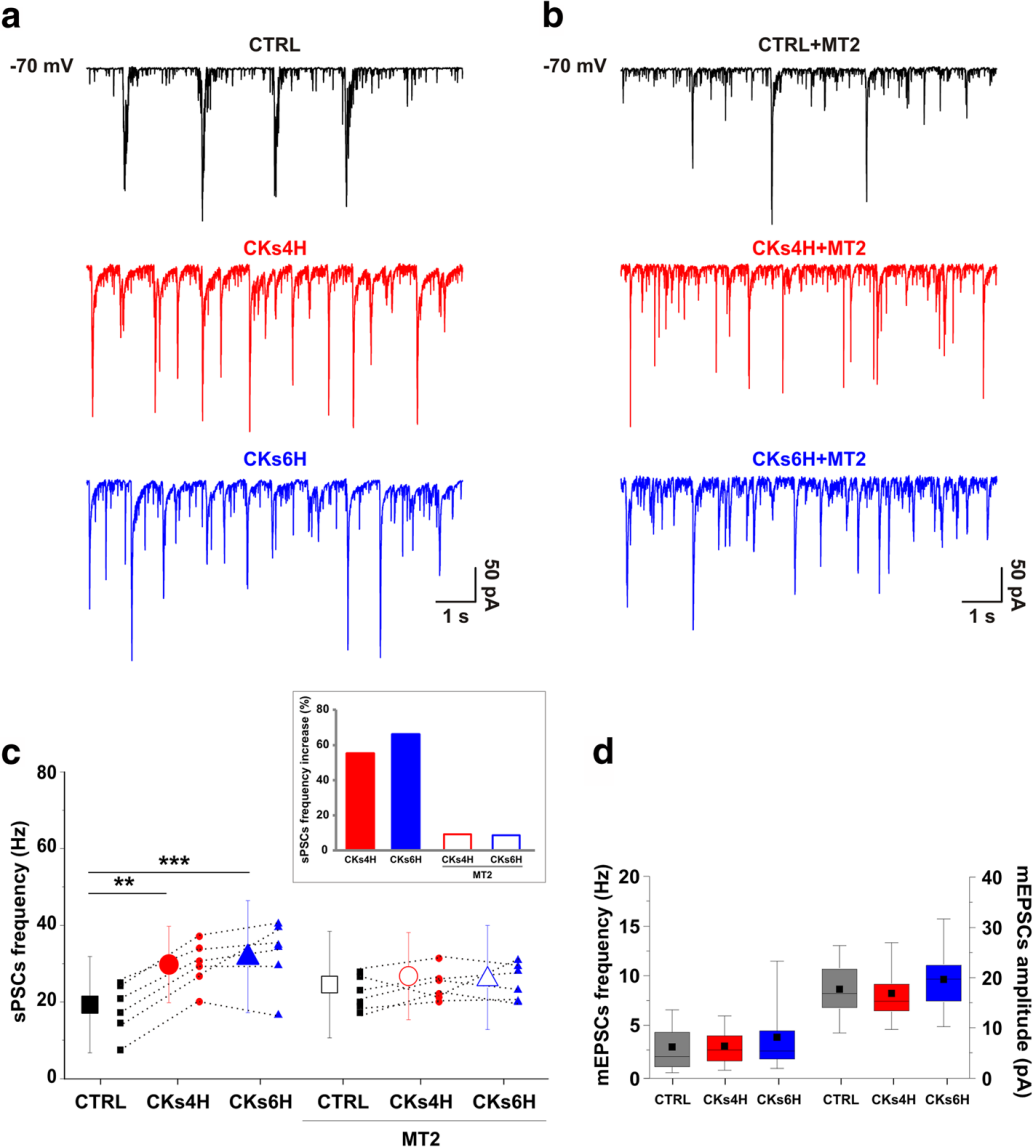
Pro-inflammatory CKs modulation of synaptic activity in organotypic spinal cord cultures is prevented by MT2. In (**a**) and (**b**) Spontaneous PSCs (sPSCs) recorded from organotypic spinal cord ventral interneurons in control (CTRL) or after CKs treatments in the absence in (**a**) or in the presence in (**b**) of MT2. Note that CKs ability to increase sPSCs occurrence is prevented by MT2 applications. **c** The plot summarizes the frequency of sPSCs prior and after CKs incubation, note the significant increase in network activity, and the frequency of sPSCs prior and after CKs incubation in the presence of MT2, note the absence of significant changes in network activity. Small symbols depict data for six different culture series (between 5 and 8 cells each); larger symbols report mean values. In the inset: the frequency of sPSCs is expressed in respect to each control group, note the increase by 55.27% in CKs4H and by 65.94% in CKs6H. Virtually no increases were observed in MT2: 9.20% in CKs4H + MT2 and 8.63% in CKs6H + MT2. **d** Box-plots summarize the mEPSC event frequency and amplitude values in control or after CKs treatments. Note that CKs applications did not affect these parameters. See the Additional file 1: Figure S1 for mEPSCs in MT2 control and MT2 CKs-treated organotypic slices

We triggered the neuro-inflammatory stress by incubating organotypic cultures with a cocktail of CKs. To directly assess the presence of changes at the level of spinal network activity we patch clamped visually identified ventral interneurons and compared, under voltage clamp mode, the emergence of heterogeneous spontaneous post-synaptic currents (sPSCs; Fig. 2a) between control (CTRL; *n* = 48) and treated cultures (CKs4H and CKs6H; *n* = 32 and *n* = 34, respectively). Organotypic spinal slices display prominent spontaneous electrical activity in the ventral, premotor area [13, 14]. To enable a meaningful comparison of the shifts in communication dynamics in networks exposed to CKs, we selected the 2 WIV stage, where neurons are known to exhibit an intense synaptic activity [13, 14]. In all culture groups, sPSCs were represented by heterogeneous inward currents of variable amplitudes (Fig. 2a and b). CKs treatments did not affect neuronal passive membrane properties (see Methods), however both CKs incubation protocols significantly increased the frequency of sPSCs. The plot in Fig. 2c shows (small symbols) data for six different culture series (between 5 and 8 cells in CTRL and CK4H or CK6H for each series), and the difference in mean values, reported as larger symbols, is statistically significant (19.1 ± 12.7 Hz CTRL; 29.6 ± 10.0 Hz CKs4H; 31.7 ± 14.7 Hz CKs6H; **P = 0.0048 CTRL vs CKs4H and **P = 0.0002 vs CKs6H, two-way ANOVA). This result is in agreement with previous works reporting the ability of pro-inflammatory CKs, such as TNF-α or IL-1β, to enhance synaptic transmission in spinal cord acute slices [17–19]. However, differently from other studies [20, 21], the neuroinflammatory milieu did not affect the frequency and amplitude of miniature (recorded in the presence of TTX, 1 μM), pharmacologically isolated (in the presence of bicuculline 20 μM and strychnine 1 μM), AMPA-glutamate receptor-mediated excitatory PSCs (mEPSCs; CTRL *n* = 37, CKs4H *n* = 20, CKs6H *n* = 20; Fig. 2d). mEPSCs are independent of network function and should primarily help to localize observed changes in synaptic transmission to pre- and/or post-synaptic level. Our results suggest that CKs treatments were not affecting network activity by tuning excitatory synapses at the pre-synaptic level, increasing the probability of release or the number of release sites, or at the post-synaptic one, altering the properties of glutamate receptors [22].

In parallel, we tested the effects of MT2 applications prior and during CKs 4H and 6H treatments (Fig. 2b). MT2 did not affect the passive properties of CTRL spinal neurons (see Methods) that exhibited a variable and slight, although not significant (*P* = 0.3292), increase in sPSCs frequency (24.5 ± 14.1 Hz CTRL + MT2, *n* = 43), as summarized in the plot of Fig. 2c. In the presence of MT2, CKs treatments at both 4H (*n* = 40) and 6H (*n* = 40) did not further boost sPSCs frequency (26.8 ± 11.5 Hz CKs4H + MT2; 26.6 ± 12.8 Hz CKs6H + MT2). MT2 ability to control the increase in sPSCs frequency brought about by CKs treatments is summarized in the inset of Fig. 2c, where the frequency of synaptic currents is expressed as % of each control.

mEPSCs frequency and amplitude were unchanged when measured in the presence of MT2, with or without CKs (see Additional file 1: Figure S1). These results support the hypothesis that MT2 prevented the increase in network activity caused by the inflammation milieu, apparently without targeting at the pre or post-synaptic level the AMPA receptor mediated synapses.

**Fig. 3:**
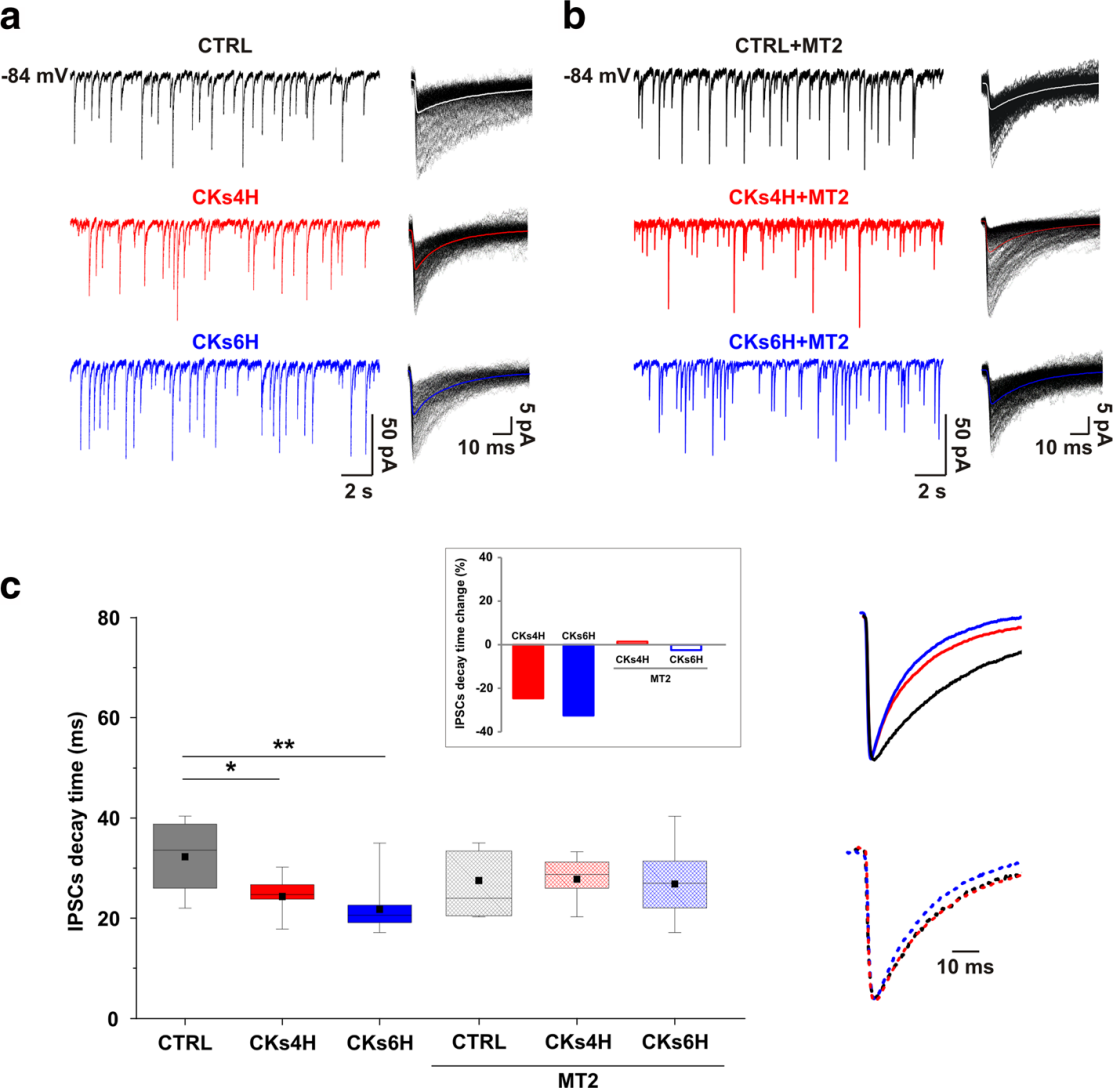
Pro-inflammatory CKs modulation of GABA_A_ receptor-mediated event time course is prevented by MT2. Representative traces of IPSC recorded from organotypic spinal slices prior and after CKs treatments in control (**a**) and in the presence of MT2 (**b**). Note that while CKs did not increase IPSC frequency, they affected IPSC time course (superimposed tracings). **c** Box-plots summarize the decay time constant values in all conditions, in the insets the scaled and superimposed average IPSC are depicted (control in black, CKs 4H in red and 6H in blue) in the absence or in the presence of MT2. Note that in the presence of MT2 no changes in the IPSC time course were detected following CKs treatments. In the inset: the τ is expressed as % in respect to each control group, note the reduction by 24.59% in CKs4H and by 32.48% in CKs6H. Virtually no decreases were observed in MT2: slight increase by 1.12% in CKs4H + MT2 and slight reduction by 2.44% CKs6H + MT2. See the Additional file 2: Figure S2 for GABAergic PSCs in the absence or in the presence of MT2

In the next set of experiments we explored fast Cl^−^ mediated synaptic transmission and, because of the relatively low frequency of glycinergic events at this developmental stage in culture [13], we compared CTRL (*n* = 16) vs CKs treated GABA_A_ receptor-mediated synaptic events (IPSCs), recorded in the presence of CNQX (10 μM), APV (25 μM) and strychnine (1 μM). Upon CKs treatments at 4H (*n* = 13) and 6H (*n* = 11) IPSC frequency (4.6 ± 3.0 Hz CTRL; 4.9 ± 2.3 Hz CKs4H; 4.7 ± 3.1 Hz CKs6H) and amplitude (20.1 ± 8.9 pA CTRL; 20.7 ± 9.5 pA CKs4H; 25.2 ± 13.6 pA CKs6H) were not changed by the inflammatory conditions in control or in the presence of MT2 (sample tracings in Fig. 3a and b; box plots in Additional file 2: Figure S2; CTRL + MT2 *n* = 16, CK4H + MT2 *n* = 14, CK6H + MT2 *n* = 18).

Superimposed IPSCs in Fig. 3a (right), show that their decay time constant (τ) becomes progressively shorter with CKs treatments. In Fig. 3c the box plot summarizes the measured τ values (32.3 ± 7.8 ms CTRL; 24.3 ± 4.7 ms CKs4H; 21.8 ± 5.6 ms CKs6H; *P = 0.020 CTRL vs CKs4H, **P = 0.0014 CTRL vs CKs6H, two-way ANOVA; scaled IPSCs average are superimposed, top). The absence of a significant correlation between IPSC rise time vs decay time values (CTRL *r* = 0.3693; CKs4H *r* = −0.1897; CKs6H *r* = 0.3419; CTRL + MT2 *r* = 0.3629; CKs4H + MT2 *r* = 0.4652; CKs6H + MT2 *r* = 0.4420; plots in Additional file 2: Figure S2) suggests that differences in recording conditions, location of synapses or electronic filtering are unlikely to have affected our observations. We extend our characterization to the properties of miniature IPSCs (mIPSCs; recorded in the presence of TTX). The results in this group of cells confirm that upon CKs treatments mIPSCs differ in their decay kinetics (45.0 ± 8.6 ms CTRL; 31.2 ± 10.4 ms CKs4H; 21.9 ± 2.1 ms CKs6H; *P = 0.024 CTRL vs CKs6H, *n* = 4 for each of the three groups, one-way ANOVA; Additional file 3: Figure S3) in a manner similar to that of spontaneous IPSCs. In the presence of MT2, IPSCs show comparable τ in all CKs treatments, as exemplified by the superimposed events in Fig. 3b. The τ values are summarized in the box plot of Fig. 3c (27.5 ± 7.7 ms CTRL + MT2, 27.8 ± 4.7 ms CKs4H + MT2, 26.8 ± 7.2 ms CKs6H + MT2; scaled IPSCs average are superimposed, bottom). The changes in GABAergic τ induced by CKs treatments are summarized in the inset of Fig. 3c, reported as % of changes in respect to each control.

We can hypothesize that the MT2 was actually able to protect inhibitory synapses from pro-inflammatory stimulation.

### Both CKs and MT2 are not interfering with spinal neurons excitability

**Fig. 4:**
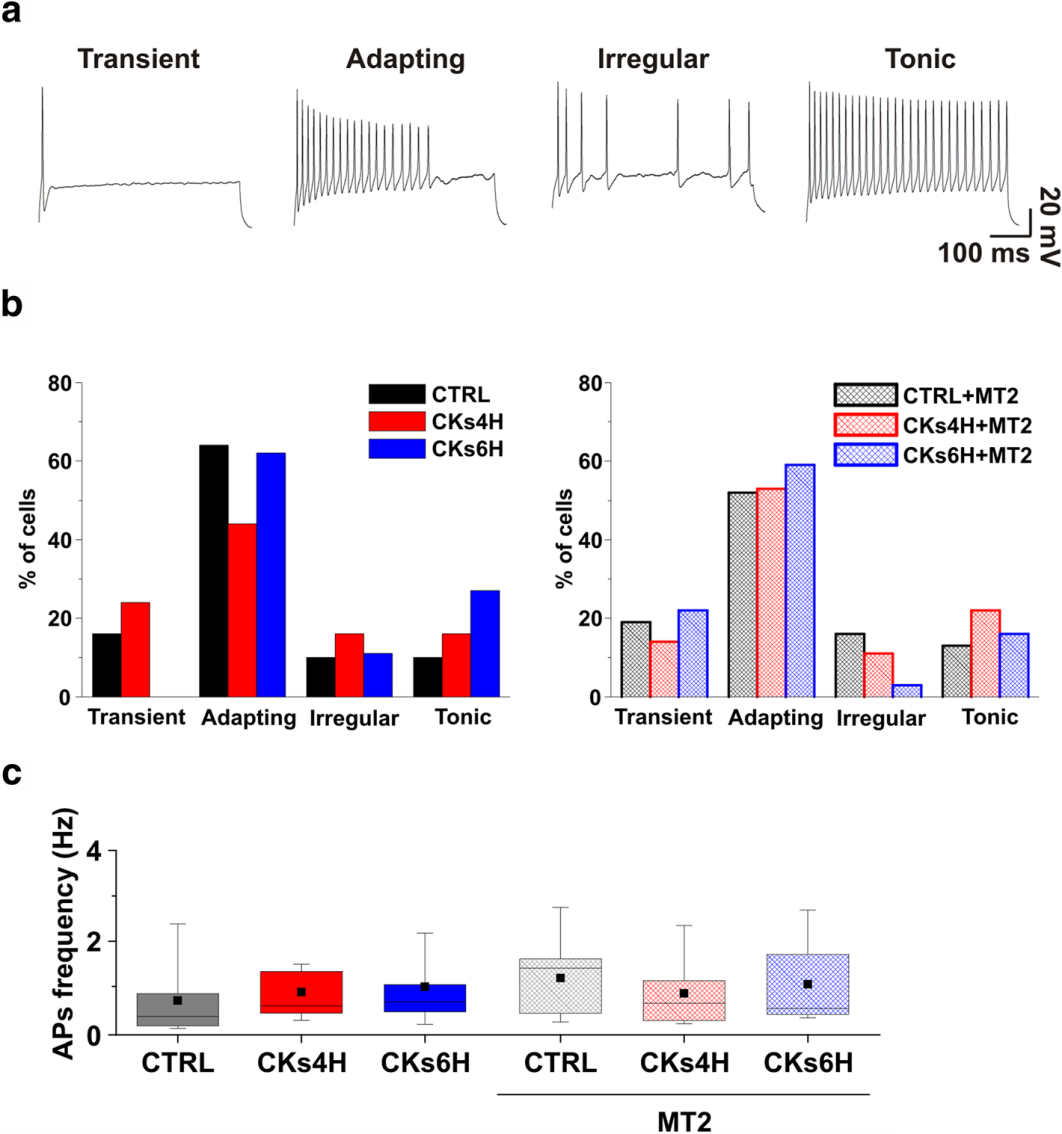
Pro-inflammatory CKs and MT2 do not alter neuronal excitability in organotypic spinal slices. **a** Discharge patterns of ventral interneurons. The 500 ms depolarizing current commands induced different discharge patterns that identified four cell categories: transient, adapting, irregular and tonic. **b** Bar-charts illustrate the probability distribution (expressed as percentage of sampled population) of each cell type in the various conditions in the absence (left) or in the presence (right) of MT2. **c** Box-plots illustrate the absence of changes in the frequency of spontaneous action potentials in all conditions tested

To elucidate the neuronal mechanisms mediating neuro-inflammatory increase in spinal activity, we addressed whether CKs changed neuronal excitability. In current clamp mode, recorded interneurones did not differ in terms of resting membrane potential and firing threshold (see Methods). Ventral interneurons in organotypic slices have been identified on the basis of their discharge patterns [13]. We identified four different classes of interneurons on the basis of their firing pattern (Fig. 4a) [13, 23–26]: ‘transient’ cells, that generated a single AP only; ‘adapting’ cells, that discharged an early burst of APs followed by adaptation; ‘irregular’ cells without discernible pattern of AP discharge; ‘tonic’ cells, that continuously fired APs without apparent accommodation. A fifth category (i.e. ‘delay’ cells, that generated APs after a lag) [13] was rarely (< 4%) observed and thus was not quantified in these series of experiments.

We used depolarizing current steps (0.1 and 0.2 nA amplitude) [13] to induce the firing patterns. The histograms depicted in Fig. 4b report the similar distribution of discharge patterns in CTRL and CK4H (CTRL 16% transient, 64% adapting, 10% irregular, 10% tonic, *n* = 31; CKs4H 24% transient, 44% adapting, 16% irregular, 16% tonic, *n* = 25) and such a distribution was not affected by MT2 treatments (CTRL + MT2 19% transient, 52% adapting, 16% irregular, 13% tonic, *n* = 31; CKs4H + MT2 14% transient, 53% adapting, 11% irregular, 22% tonic, *n* = 36). The only different value observed was the virtual absence in CKs6H of transient firing cells, on the contrary well represented in CKs6H + MT2 (CKs6H 0% transient, 62% adapting, 11% irregular, 27% tonic, *n* = 26; CKs6H + MT2 22% transient, 59% adapting, 3% irregular, 16% tonic, *n* = 37).

The box plots in Fig. 4c quantify the frequency of spontaneous APs, and also in this case no differences were detected among CTRL and CKs treated (CTRL 0.7 ± 0.8 Hz, *n* = 14; CKs4H 0.9 ± 0.8 Hz, *n* = 14; CKs6H 1.0 ± 1.1 Hz, *n* = 12) or CTRL + MT2 and CKs + MT2 (CTRL + MT2 1.3 ± 0.9 Hz, *n* = 14; CKs4H + MT2 0.8 ± 0.8 Hz, *n* = 18; CKs6H + MT2 1.0 ± 1.0 Hz, *n* = 13).

These results suggest that cell excitability was not affected by CKs or MT2.

### Inflammatory stress modifies cytokine and chemokine production and induces astrogliosis

**Fig. 5:**
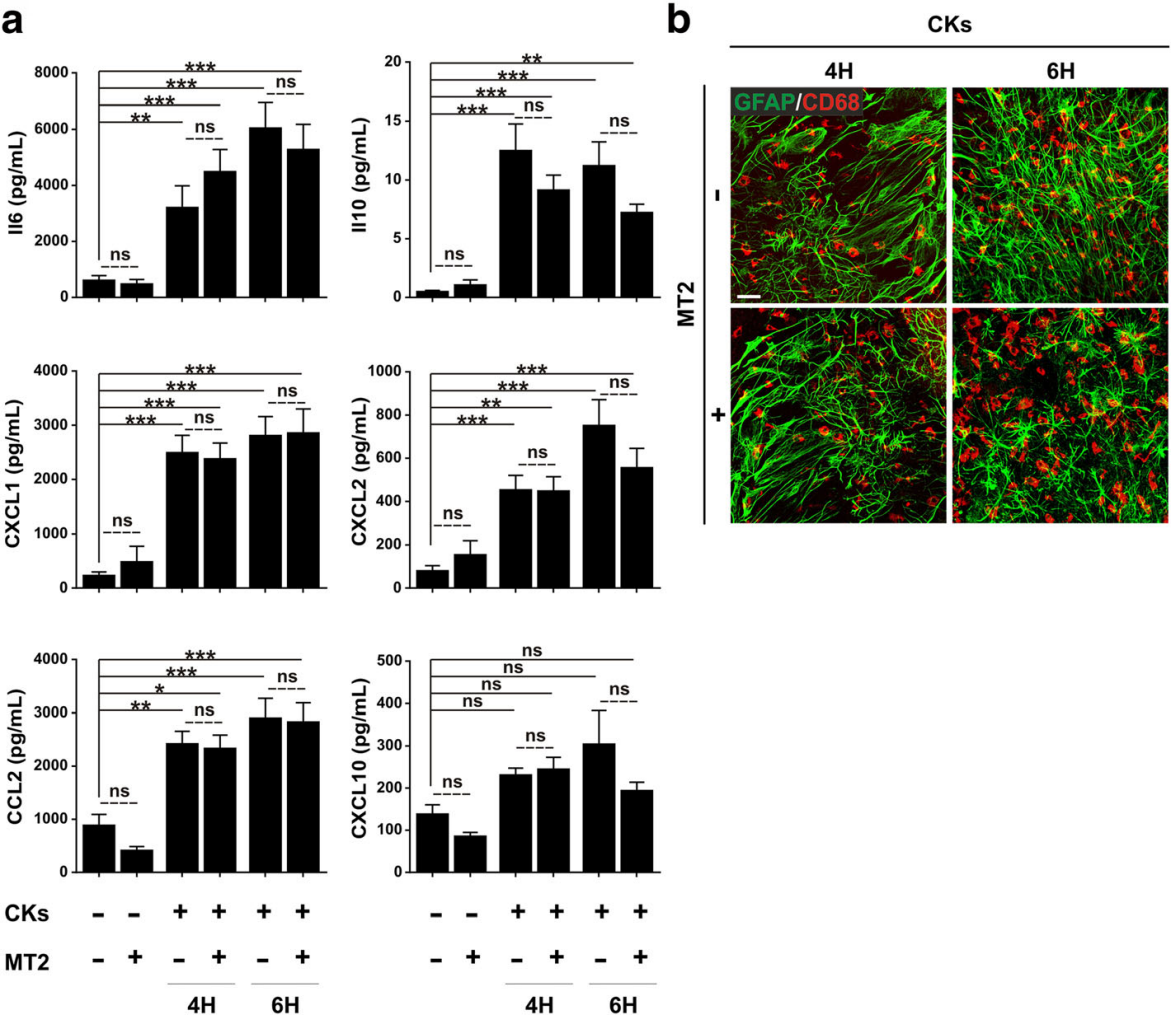
Effects of pro-inflammatory stress on soluble factor production and on astro/microglia morphology in organotypic spinal slices. **a** Production of cytokines (IL6; IL10) and chemokines (CXCL1; CXCL2; CCL2; CXCL10) determined by Milliplex assay in organotypic culture supernatants after stimulation with the pro-inflammatory cytokine cocktail (CKs) for 4 or 6 h in the presence or absence of MT2. Column graphs report mean values ± SEM of 25 independent experiments. The pro-inflammatory stimulus significantly increases the release of IL6, IL10, CXCL1, CXCL2 and CCL2 compared to control (first column), both after 4 and 6 h of treatment. MT2 treatment did not alter the soluble factor production. **b** Immune fluorescence staining for astrocytes (GFAP, green) and microglia (CD68, red). The pro-inflammatory stress (CKs) induces astrocyte spreading and microglia activation, as indicated by the amoeboid shape. MT2 treatment did not affect these cellular patterns. The pictures show one field representative of 5 randomly analyzed ones (one experiment representative of 5 independent ones). Calibration bar 20 μm

We evaluated the production of cytokines and chemokines by organotypic spinal cord slices in response to pro-inflammatory stress. The summarizing plots of Fig. 5a show that the exposure to Th1 cytokine cocktail significantly increases the release of IL6 and IL10, key players in regulating inflammation, as well as the release of CXCL1, CXCL2 and CCL2, chemokines implicated in the recruitment of innate immune cells (*0.01 < *P* < 0.05; **0.001 < *P* < 0.01; ***P < 0.001; one-way ANOVA). CXCL10, a T cell chemoattractant, is not affected by these treatments. MT2, independently on the inflammatory stimulation, does not exert any effect in the release of any analyzed soluble factor. As expected, the altered cytokine profile induced by pro-inflammatory stress is accompanied by marked astrogliosis and microglia activation, qualitatively illustrated in the example of Fig. 5b (compare with Fig. 1b).

### MT2 modulates p38 MAPK activation induced by cytokine stress

**Fig. 6:**
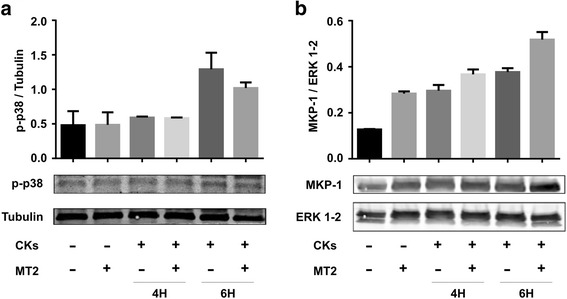
Effects of MT2 on MAP Kinase pathway in organotypic spinal slices. Organotypic cultures were stimulated with pro-inflammatory CKs cocktail for 4 or 6 h, in the presence or in the absence of MT2. **a** Slices lysates were blotted with rabbit anti-P-p38 and mouse anti-Tubulin as loading control. Bar-charts represent the data of densitometric analysis and are expressed as the ratio between phospho-p38 and Tubulin proteins. **b** Slices lysates were blotted with rabbit anti-MkP-1 and rabbit anti-ERK 1/2 as loading control. The bar-charts represent the data of densitometric analysis and are expressed as the ratio between MKP-1 and ERK 1/2 proteins

It is generally accepted that many highly conserved serine/threonine mitogen-activated protein kinases (MAPK), including p38 MAPK, are activated in response by environmental and cellular stresses including CKs [15]. We investigated the interplay between MT2 treatment and p38 MAPK activation in organotypic slices undergoing pro-inflammatory cytokines incubation. Fig. 6a shows the result of western blot analysis with specific antibody to phospho-p38 MAPK performed in lysates of organotypic slices subjected to CKs stimuli. The data show a de-phosphorylation of p38 MAPK in MT2 treated slices. To better understand the modulation of p38 MAPK activation elicited by MT2, we tested whether MKP-1, a phosphatase highly specific for p38 MAPK and also for JNK, could have a role in this setting. Figure 6b shows that, at 6H CKs treatment, MT2 caused an increase in MKP-1 expression. Taken together these data suggest that MT2 has trophic effects on slices undergoing metabolic derangement induced by pro-inflammatory cytokines.

## Discussion

The dogma of the CNS immune-privilege is progressively weakening. In the last decade, an increasing amount of results suggested that, notwithstanding its being surrounded by the BBB, the CNS is a highly immunological active organ, characterized by complex immune cell activation [27] resulting in beneficial (protective) as well as harmful (degenerative) responses [28].

Inflammation, synaptic transmission and neuronal damage are ultimately linked [7]. In this work we have investigated pro-inflammatory CKs effects in spinal microcircuits developed in organotypic cultures containing microglia, astrocytes and neurons, focusing on synaptic activity and measuring soluble factor production. Last, we exploited this model to demonstrate the synaptic protective role of neurotrophins by treating the culture with a non-peptidic mimetic molecule (MT2) that binds TrkA/B receptors.

We selected a CKs cocktail able to mimic an inflammatory reaction that spreads in CNS containing IL-1β, well known determinant of neuropathy [3, 7], TNF-α, that is ubiquitary present during Th1/Th17 mediated inflammatory reactions, and GM-CSF, key cytokine responsible of pro-inflammatory effects in the CNS of MS animal models [29]. GM-CSF receptors are expressed in microglia, therefore the use of this soluble factor allows targeting resident microglial cells, present within the organotypic spinal explanted tissue. Indeed, one of our aims was that to address synaptic function in CNS circuits in the presence of astrocytes and microglia exposed to CKs. We used factors known to be released in EAE and able to directly and indirectly (via activation of resident cells) target neuronal functions. We adopted relatively acute treatments, which triggered inflammatory responses, without affecting neuronal membrane properties or inducing direct neurotoxicity, yet still able to alter synaptic transmission. This protocol has allowed unmasking subtle early changes in GABAergic synaptic currents, a significant player in the excitation/inhibition balance of pre-motor outputs.

### Pro-inflammatory CKs tune the excitability of organotypic pre-motor circuit

In organotypic spinal tissue we confirmed the presence of heterogeneous neuroglial cells after 2 weeks of in vitro growth [9], and we further documented the expression and cell-localization of TrkA/B receptors (see below). In the present study, we have used a CKs cocktail and set a tissue exposure protocol to these agents that did not alter TrkA/B receptor expression, however such agents induced a reliable release of cytokines and chemokines, mostly due to the local generation and delivery of inflammatory factors. In fact, upon CKs stimulus, we reported clear inflammatory tissue reactivity expressed as IL6 and monocytes recall-chemokines release, likely requiring the involvement of the heterogeneous cell types present in the slice culture. Our experimental model is ideally suited to dissect spinal resident cells ability in modulating local synapses, in the absence of any contributions from the peripheral infiltrating cells, this mimics early phases of multiple sclerosis where immune cells in brain perivascular spaces activate and produce inflammatory CKs that easily diffuse before cells enter the CNS [30]. In particular, CNS cultured explants allow investigating complex synaptic networks preserving the basic cytoarchitecture of the original CNS area [31], but, differently from acute slices, long term culturing allows the complete recovery from the altered metabolic state caused by the tissue dissection and promotes the effective clearance from the tissue debris, due to the slicing procedure [32, 33].

We recorded from interneurons to assess how the pro-inflammatory stress may regulate the ventral (pre-motor) [14] circuit activity, and this was done within a time-frame where the inflammatory pathological process did not progress to the extent of excitotoxicity. The lack of clear cell damage was supported by the absence of changes in the values of resting membrane potential, input resistance, cell capacitance and AP threshold in the treated neurons in respect to control ones [34–36].

The present data show that, after 4 and 6 h CKs treatments, the frequency of sPSCs was increased. An increased synaptic transmission is a described feature of pro-inflammatory stress in neural circuits, reported form ex-vivo CNS slices isolated from EAE mouse models or from healthy slices exposed to cerebrospinal fluid from multiple sclerosis patients [3, 5, 37] as well as from acute spinal cord slices transiently exposed to various pro-inflammatory molecules [17–19]. Such increases in synaptic transmission have been attributed to the up-regulation of the glutamatergic system [3, 5, 37], to the down-regulation of inhibitory transmission [17, 18, 38] or to altered cell excitability [19, 39]. In spinal cultured slices we did not detect the signatures of any of these mechanisms. To understand the reason for the observed boost in spinal network activity, we examined the miniature excitatory currents [22] that did not suggest the presence of changes in pre-synaptic release probability and in the number of synaptic contacts or in post-synaptic receptor sensitivity in glutamatergic synapses due to CKs exposure, we also excluded alterations in single cell excitability. We equally ruled out variations in the general distribution of firing patterns [13, 19] and in the frequency or amplitude of IPSCs.

We focused our attention to the fast Cl^−^-mediated neurotransmission due to GABA_A_ receptor activation, recently indicated as a specific CKs target in spinal circuits [17, 19, 39]. Intriguingly, we found that both IPSCs and mIPSCs from 4 and 6 h CKs slices decayed faster than those from controls, in the absence of any other changes, including differences in release synchronization [14]. This is, to our knowledge, the first time that such a modulation of GABAergic activity is reported due to pro-inflammatory CKs exposure. We did not further investigate in the present work the mechanisms responsible for the altered kinetic properties of GABAergic receptors and therefore the IPSC time course, which can involve differences in the intracellular chloride concentration [40, 41] as well as changes in the receptor subunit composition [42].

Regardless the mechanisms involved, our experimental conditions unmasked a sophisticated and specific synaptic regulation during inflammatory states that may contribute to the increase pre-motor circuit excitability via network-mediated mechanisms. In fact, the faster decaying GABAergic PSCs will lead to weaker and shorter-lasting inhibition, resulting in a reduction in charge transfer in the range 28–35%, perhaps associated with increased network activity. This hypothesis is in agreement with the MT2 ability to control both the CK-mediated increase in network activity and the reduction in IPSC decay. It is tempting to speculate that the different CKs cocktail, the duration of the exposure used here, longer than in the previous studies on acute spinal slices [17, 19, 39], and, in particular, the presence of resident cells, are all factors ultimately responsible of this divergence from previous results. In addition, when compared to acute slicing, the presence of full metabolic recovery from dissection in organ cultures, might also play a significant role.

Interestingly, in previous reports, in dissociated cultures exposed to inflammatory cues [38], the involvement, during chronic EAE phases, of perturbed GABAergic transmission, was suggested together with the need of alternative neuroprotective strategies.

### MT2 protective effect on spinal organotypic cultures

With the aim to restore spinal network activity following the neuroinflammatory treatment, we turned our attention to NGF. NGF is a well-known regulator of neuronal differentiation and plasticity [43]. More recently, NGF has been associated to inflammation and autoimmune diseases. NGF, via TrkA receptors, down-regulates inflammatory CKs production while inducing the release of anti-inflammatory mediators [44]. Moreover, administration of NGF in vivo in EAE animals delays the onset of clinical symptoms and prevents the full development of EAE lesions [45–48]. Despite these exciting reports, the therapeutic use of neurotrophins is a daunting task, due to their limited ability to diffuse in tissues [15]. Therefore, the design of small molecules able to interact with neurotrophins receptors is of great therapeutic interest. In our cultures, TrkA is specifically expressed in DRG and other neurons; on the other hand, we found TrkB expression also in astrocytes. TrkB expression in cultured DRG was not unexpected [49], reviewed in [50]; the finding of TrkB expression in astrocytes is, although surprising for cultured tissue, reminiscent of the complexity of the CNS in vivo. In fact, in the adult rodent spinal cord TrkB expression has been described in reactive astrocytes [51–53]. We did not detect TrkB in CD68+ microglia, present in our culture mostly in amoeboid (activated) form after CKs treatments, in agreement with a previous report on the spinal cord [54–56]. Notwithstanding TrkB expression in the organotypic GFAP-positive astrocytes, (probably the main source of IL-6), the sole addition of MT2 did not modify CK profile in the supernatants, neither directly nor by neuron mediated immune modulation [57]. However, MT2 controlled the CKs electrophysiological signature, namely the induced increase in the sPSCs frequency and the modulation of IPSC time course. To note, MT2 provoked a slight increase in synaptic activity per se, although not significant, and one could argue that in this manner the CKs potentiating effect was simply occluded. Against this interpretation, we also reported the efficacy of MT2 in preventing IPSC decay time modulation. Previous observations [15] suggested the possibility of relatively fast transduction events, after engaging the tyrosine kinase receptors; such mechanisms may explain the short time (no more than 4 h) needed in order to observe the MT2 counteracting effect on electrophysiological alterations. We also reported a trend of decrease in p-p38 accompanied by an increase in MKP-1. These results are, although preliminary, suggestive of such pathways involvement in rescuing GABAergic PSCs.

In conclusion, we developed an important tool for the study of spinal cord alterations induced by inflammation, that takes into account the role of resident cells: neuronal and not neuronal populations, this tool allowed us to test a potential therapeutic molecule.

## Methods

### Preparation of spinal cord organotypic cultures and neuroinflammation treatments

Briefly, organotypic slice cultures of spinal cord and dorsal root ganglia (DRG) were obtained from mouse embryos (C57BL/6 J of either sex) at days 12–13 of gestation as previously described [13, 14, 58, 59]. Pregnant mice were sacrificed by CO_2_ overdose and fetuses delivered by caesarean section. Isolated fetuses were decapitated and their backs were isolated from low thoracic and high lumbar regions and transversely sliced (275 μm) with a tissue chopper. After dissecting the spinal cord slices from the surrounding tissue, slices were embedded into a thick matrix obtained by chicken plasma (Rockland) and thrombin (Sigma) clot. Slices were cultured in plastic tubes with 1 mL medium [14]. The tubes were kept in a roller drum rotating 120 times per hour in an incubator at 37 °C in the presence of humidified atmosphere, with 5% CO_2_. Experiments were performed on spinal cultures at 2-weeks in vitro (WIV). The day of the experiment, organotypic spinal cord slices were incubated with standard medium (Control, CTRL) or, for 4 or 6 h (4H and 6H), with a cocktail of the following mouse recombinant cytokines: TNF-α (R&D Systems, #210-TA/CF), IL-1β (R&D Systems, #M15330), and granulocyte-macrophage colony-stimulating factor (GM-CSF; R&D Systems, #P04141), 10 ng/mL each, in order to induce an inflammatory state (Hanisch, 2002). CKs were removed after 4H and 6H, prior to electrophysiological recordings. In sister cultures, controls and the incubation with the CKs cocktail was done in the presence or absence of MT2, a NGF mimetic non-peptidic TrkA and TrkB ligand, kind gift from MimeTech srl, Rome, Italy. MT2 (10 μM for 4H) was tested in the same three conditions (CTRL, CKs4H and CKs6H; in CKs6H condition, MT2 was added after the first 2H of the CKs treatment).

### Immunofluorescence and microscopy

Organotypic cultures were fixed in 4% formaldehyde (prepared from fresh paraformaldehyde; Sigma) in PBS for 1 h at room temperature (RT; 20–22 °C), washed in PBS and incubated at RT for 1 h in blocking/permeabilizing solution consisting of 3% FBS and 3% BSA (Sigma) and 0.3% Triton-X 100 (Sigma) in PBS. Then, slices were incubated over night at 4 °C with a combination of the following primary antibodies, diluted in blocking/permeabilizing solution: mouse monoclonal anti-glial fibrillary acidic protein, (GFAP; Sigma, #G3893, RRID:AB_477010, 1:200); rabbit polyclonal anti-ionized calcium-binding adapter molecule 1, (Iba1; Wako, #019–19,741, RRID:AB_839504, 1:400); rat monoclonal CD68 (Abcam, #ab53444, RRID:AB_869007, 1:200), rabbit polyclonal against Tropomyosin receptor kinase A (TrkA), (Santa Cruz, #SC-14024, RRID:AB_2298807, 1:200); rabbit monoclonal against Tropomyosin receptor kinase B (TrkB), (Cell Signaling Technologies, #4607S, RRID:AB_2155128 1:100); mouse monoclonal anti-Neurofilament H Non-Phosphorylated (SMI-32; EMD-Millipore, #NE1023, RRID:AB_2043449, 1:200). Subsequently slices were PBS-washed and incubated with secondary antibodies diluted in blocking/permeabilizing solution for 2 h at room temperature (RT) in the dark. The secondary antibodies we used were: Alexa 635 goat anti-mouse (Invitrogen, #A31574, RRID:AB_2536184, 1:250); Alexa 488 goat anti-mouse (Invitrogen, #A11001, RRID:AB_2534069, 1:400); Alexa 546 goat anti-mouse (Invitrogen, #A11003, RRID:AB_141370, 1:400); Alexa 488 donkey anti-rabbit (Abcam, #ab150061, RRID:AB_2571722, 1:300). Samples were PBS-washed and mounted on glass slides with ProLong® Diamond Antifade Mountant with DAPI (Thermo Fisher Scientific). Stained samples were examined with 20× and 40× magnification on a Laser Scanning Confocal Microscopy (LSM 5109 Meta, ZEISS); sections were acquired at different focal planes every 1 μm. The image analyses were performed using the ImageJ software (http://rsbweb.nih.gov/ij/).

### Western blot analysis

Organotypic slices (*n* = 5 for each experimental condition) were lysed with RIPA buffer (50 mM Tris-HCl, pH 7.4; 150 mM NaCl; 2 mM EDTA; 1 mM NaF; 1 mM sodium orthovanadate, 1% NP-40) in the presence of phosphatase inhibitor cocktail 2 and 3, protease inhibitor cocktail (Sigma Aldrich) and centrifuged at 12.000 r.p.m. for 15 min. 40 μg of total proteins were loaded into SDS-PAGE and blotted onto nitrocellulose filters (GE Healthcare, Fairfield, CT, USA). Membranes were stained with rabbit anti-TrkA, anti-TrkB, anti-MKP-1, anti-ERK ½, anti-P-p38 (Cell Signaling), mouse anti-GAPDH (Cell Signaling), mouse anti-α-tubulin (Santa Cruz Biotechnology); all the antibodies were used at 1:1000, final dilution. HRP-coniugated anti-rabbit IgG (GE Healthcare) or HRP-conjugated anti mouse IgG (Santa Cruz Biotechnology) were used as secondary antibodies at 1:2000 final dilution. The reactions were visualized by the ECL detection system as recommended by the manufacturer (GE Healthcare).

### Electrophysiological recordings

For patch clamp recordings (whole-cell) a coverslip with the spinal culture was positioned in a recording chamber, mounted on an inverted microscope (Nikon Eclipse TE200 and Nikon Eclipse Ti-U), and superfused with a standard saline solution containing (in mM): 152 NaCl, 4 KCl, 1 MgCl_2_, 2 CaCl_2_, 10 HEPES and 10 glucose. The pH was adjusted to 7.4 with NaOH (osmolarity 305 mOsm). Visually identified ventral interneurons were patched with pipettes (4–7 MΩ) filled with a solution of the following composition (in mM): 120 K gluconate, 20 KCl, 10 HEPES, 10 EGTA, 2 MgCl_2_, 2 Na_2_ATP. The pH was adjusted to 7.3 with KOH (295 mOsm). All recordings were performed at RT.

Under voltage clamp configuration, the voltage values indicated are corrected for the liquid junction potential (−14 mV) [14] if not otherwise indicated. Series resistance values were < 10 MΩ enabling recordings of synaptic currents without significant distortion, and were not compensated for [13]. Recordings were performed from ventrally located spinal interneurones identified on the basis of previously reported criteria [13, 60, 61]. Electrophysiological responses were amplified (EPC-7, HEKA; Multiclamp 700B, Axon Instruments), sampled and digitized at 10 kHz with the pCLAMP software (Axon Instruments) for offline analysis.

In voltage-clamp recordings, single spontaneous post-synaptic currents (sPSCs) were detected by the use of the AxoGraph X (Axograph Scientific) event detection program [62] and by the Clampfit 10 software (pClamp suite, Axon Instruments). On average, ≥ 400 events were analysed from each cell in order to obtain mean kinetic and amplitude parameters. From the average of these events we measured the rise time defined as the 10–90% time needed to reach the peak of the synaptic current, the peak amplitude and the decay time constant (expressed as τ) by fitting a mono-exponential function.

We detected no differences in membrane capacitance (45 ± 21 pF CTRL, 46 ± 19 pF CKs4H, 48 ± 18 pF CKs6H; *n* = 55, 41, 39, respectively; and 48 ± 21 pF CTRL + MT2, 43 ± 16 pF CKs4H + MT2, 46 ± 24 pF CKs6H + MT2; *n* = 53, 47, 47, respectively) and input membrane resistance (417 ± 247 MΩ CTRL, 415 ± 328 MΩ CKs4H, 424 ± 404 MΩ CKs6H; and 400 ± 354 MΩ CTRL + MT2, 430 ± 309 MΩ CKs4H + MT2, 407 ± 244 MΩ CKs6H + MT2) of ventral spinal interneurons recorded in the different conditions. GABAergic post-synaptic currents (IPSCs) were recorded at −84 mV holding potential in the presence of CNQX (10 μM; Sigma), strychnine (1 μM; Sigma) and APV (25 μM; Sigma) and tetrodotoxin (TTX; 1 μM, Latoxan) was used to isolate GABA_A_ receptor-mediated miniature events (mIPSCs). GABA_A_ receptor-mediated PSCs were fully blocked by the application of 10 μM SR-95531 (Sigma). AMPA-glutamate receptor-mediated PSCs were recorded at −70 mV holding potential in the presence of strychnine (1 μM; Sigma) and bicuculline (10 μM; Sigma), and TTX (1 μM, Latoxan) was used to isolate AMPA-glutamate receptor-mediated miniature events (mEPSCs). AMPA-glutamate receptor-mediated PSCs were fully blocked by the application of 10 μM CNQX (Sigma).

During current clamp recordings, bridge balancing was continuously monitored and adjusted [13]. Action potentials (APs) were isolated off line by setting an appropriate threshold (−34 mV). The fast (~ 3 ms duration) voltage transients that crossed this threshold were identified as APs. The spontaneous firing frequency for each neuron was calculated on a sample of at least 5 min of continuous recording at −74 mV resting membrane potential. APs threshold was experimentally determined by depolarizing current steps [63].

Induced AP discharge patterns were investigated by delivering depolarizing current steps (500 ms duration) of 0.1–0.2 nA amplitude while keeping the cells at −74 mV resting potential with steady intracellular current injection. We did not detect differences between all the conditions tested in neither interneuron resting membrane potential (−64 ± 9 mV CTRL, −65 ± 9 mV CKs4H, −63 ± 12 mV CKs6H; *n* = 21, 16, 16, respectively; and −66 ± 8 mV CTRL + MT2, −68 ± 6 mV CKs4H + MT2, −64 ± 7 pF CKs6H + MT2; *n* = 24, 21, 22, respectively), nor in the spike threshold (−53 ± 4 mV CTRL, −54 ± 5 mV CKs4H, −53 ± 5 mV CKs6H; and −51 ± 5 mV CTRL + MT2, −53 ± 4 mV CKs4H + MT2, −52 ± 3 mV CKs6H + MT2). Electrophysiological data were obtained from 20 different culture series.

### Cytokines and chemokines measurement

IL6, IL10, CCL2, CXCL1, CXCL10 and CXCL2 concentrations were measured in organotypic culture supernatants by Milliplex assay (Merck Millipore, #MCYTOMAG-70 k), using the Bio-Plex apparatus (Biorad), according to the manufacturer’s recommendations.

### Statistical analysis

All values from samples subjected to the same experimental protocols were pooled together and results are presented as mean ± S.D., if not otherwise indicated; *n* = number of neurons. Two-way analysis of variance (two-way ANOVA) and one-way ANOVA were used to determine significance when multiple groups were compared. Statistical significance was determined at *P* < 0.05.

## Additional files

Additional file 1: Miniature excitatory PSCs were not affected by MT2 prior or after CKs treatments. The box plots summarize the mEPSCs frequency (A) and amplitude (B) in control and CKs-treated organotypic slices. (TIFF 294 kb)

Additional file 2: The frequency and amplitude of GABAergic PSCs were not affected by CKs treatments in the absence of in the presence of MT2. Box-plots summarize the frequency (A) and the amplitude (B) of IPSCs prior and after CKs incubation in both the absence and the presence of MT2. (C) The plots show the absence of linear correlation between the decay time constant and rise time of IPSCs in all the conditions tested. (TIFF 777 kb)

Additional file 3: Miniature inhibitory PSCs were faster after CKs treatments. Box-plots summarize the decay time constant values of mIPSCs in all conditions (A). Note the speeding up of the event time course following CKs treatments. (TIFF 89 kb)

## Abbreviations

AP: Action potential
BBB: Blood brain barrier
CKs: Cytokines
DRG: Dorsal root ganglia
EAE: Experimental autoimmune encephalomyelitis
GFAP: Glial fibrillary acidic protein
GM-CSF: Granulocyte-macrophage colony-stimulating factor
Iba1: Ionized calcium-binding adapter molecule 1
IL-1β: Interleukin-1beta
IPSCs: GABA_A_-receptor mediated inhibitory post-synaptic currents
mEPSCs: AMPA-receptor mediated miniature excitatory post-synaptic currents
mIPSCs: GABA_A_-receptor mediated miniature inhibitory post-synaptic currents
MSC: mesenchymal stem cell
NGF: Nerve growth factor
RT: Room temperature
SMI-32: Neurofilament H non-phosphorylated
sPSCs: Spontaneous post-synaptic currents
TNF-α: Tumor-necrosis factor-alfa
TrkA: Tropomyosin receptor kinase A
TrkB: Tropomyosin receptor kinase B
TTX: Tetrodotoxin
WIV: Week in vitro
