# Supplementary figures and images for: Bridging pro-inflammatory signals, synaptic transmission and protection in spinal explants in vitro
Source: Mol Brain. 2018 Jan 15;11:3. doi: 10.1186/s13041-018-0347-x (PMC5769440; doi:10.1186/s13041-018-0347-x)

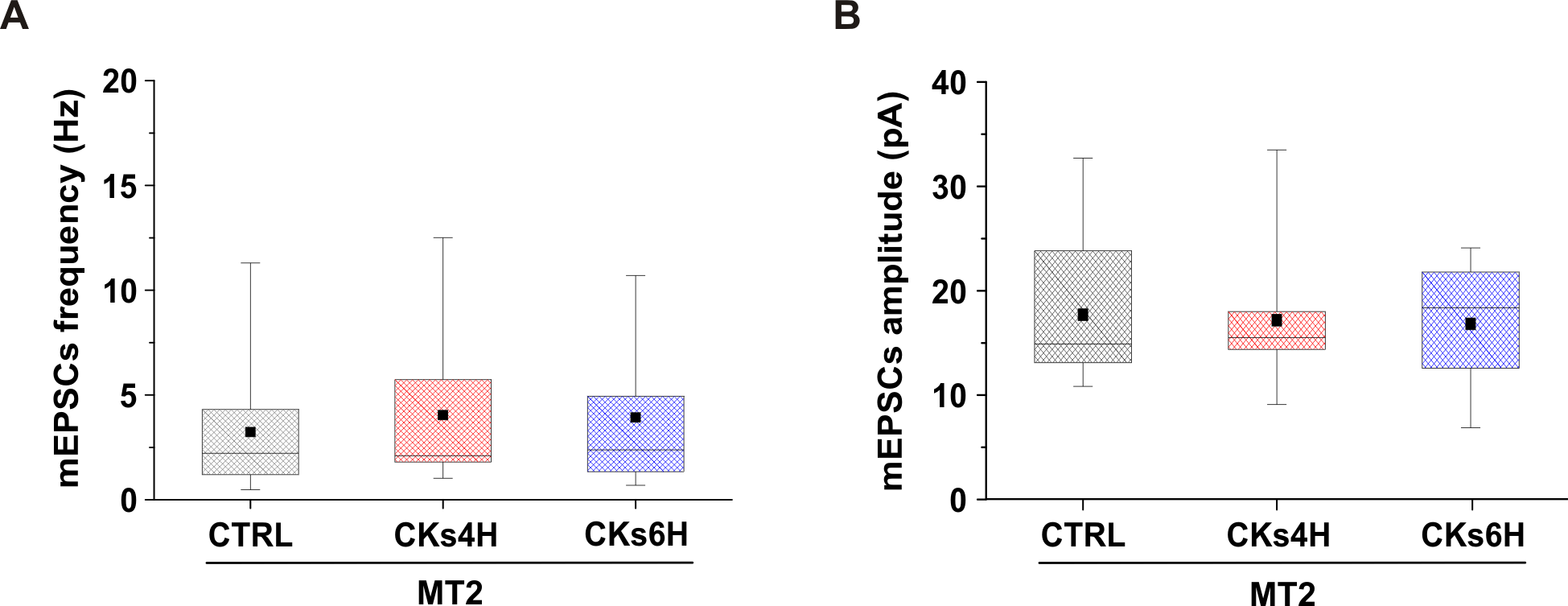

Supplement: Supplementary file 1 — Miniature excitatory PSCs were not affected by MT2 prior or after CKs treatments. The box plots summarize the mEPSCs frequency (A) and amplitude (B) in control and CKs-treated organotypic slices. (TIFF 294 kb) [file 13041_2018_347_MOESM1_ESM.tif]

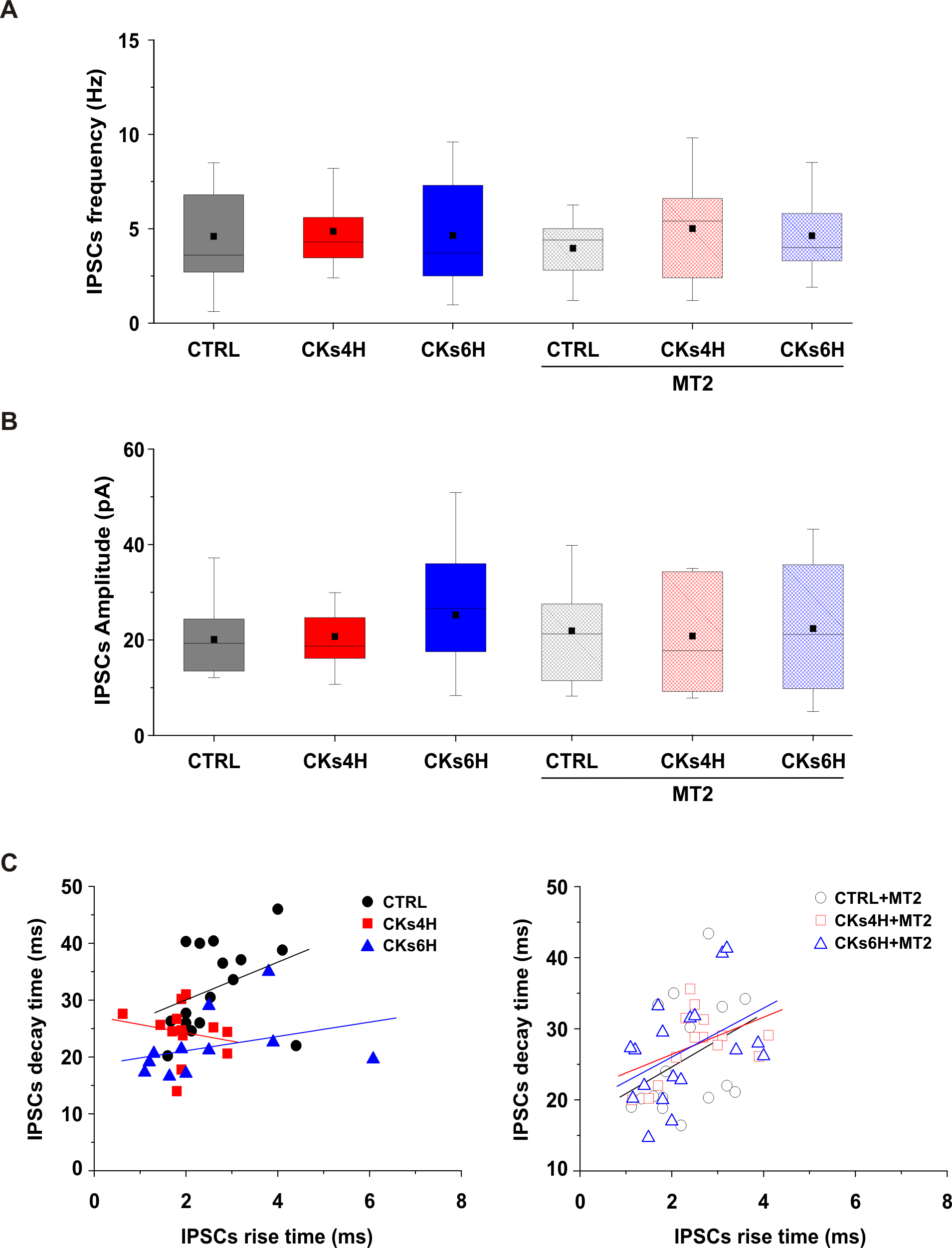

Supplement: Supplementary file 2 — The frequency and amplitude of GABAergic PSCs were not affected by CKs treatments in the absence of in the presence of MT2. Box-plots summarize the frequency (A) and the amplitude (B) of IPSCs prior and after CKs incubation in both the absence and the presence of MT2. (C) The plots show the absence of linear correlation between the decay time constant and rise time of IPSCs in all the conditions tested. (TIFF 777 kb) [file 13041_2018_347_MOESM2_ESM.tif]

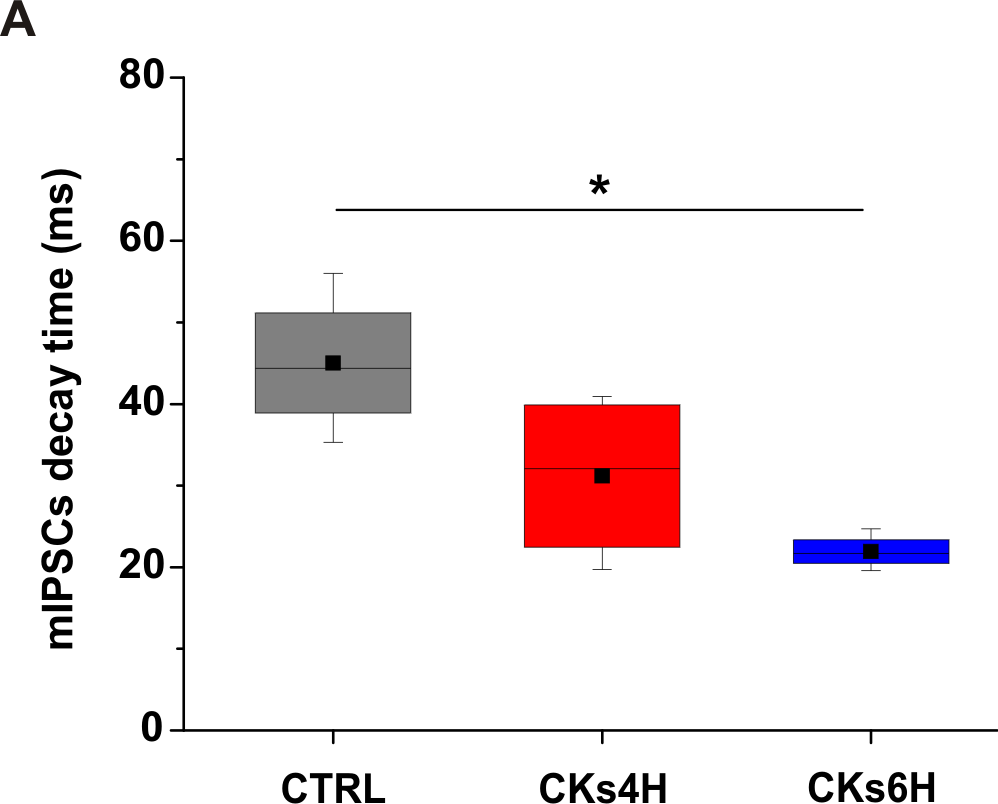

Supplement: Supplementary file 3 — Miniature inhibitory PSCs were faster after CKs treatments. Box-plots summarize the decay time constant values of mIPSCs in all conditions (A). Note the speeding up of the event time course following CKs treatments. (TIFF 89 kb) [file 13041_2018_347_MOESM3_ESM.tif]
